# Lessons learned from engaging communities for Ebola vaccine trials in Sierra Leone: reciprocity, relatability, relationships and respect (the four R’s)

**DOI:** 10.1186/s12889-019-7978-4

**Published:** 2019-12-11

**Authors:** Sara Dada, Gillian McKay, Ana Mateus, Shelley Lees

**Affiliations:** 1 London School of Hygiene and Tropical Medicine, Keppel Street, London, WC1E 7HT UK; 2 The Royal Veterinary College, Royal College Street, London, NW1 0TU UK

**Keywords:** Community engagement, Vaccine trials, Ebola, Sierra Leone, Disease preparedness

## Abstract

**Background:**

Building trust and engaging the community are important for biomedical trials. This was core to the set up and delivery of the EBOVAC-Salone and PREVAC Ebola vaccine trials in Sierra Leone during and following the 2014–2016 West African Ebola epidemic. Local community liaison teams (CLT) engaged with the community through public meetings, radio chat shows, and other activities, while a social science team (SST) assessed community members’ and participants’ perceptions and regularly updated the clinical team to adapt procedures to improve the acceptability and compliance of the trial. The objective of this study was to examine the community engagement (CE) program in these trials and to identify potential barriers and facilitators.

**Methods:**

Fifteen CLT and SST members participated in in-depth interviews and 23 community members attended three focus groups to discuss the Ebola vaccine trials and their experiences and perspectives of the CE activities.

**Results:**

A key aim of the CE program was to build trust between the community and the trial. Four main principles (the “four R’s”) evolved from the discussions with team members and the community that influenced this trust: reciprocity, relatability, relationships and respect. The CLT and SST ensured **reciprocal** communication between the trial team and the community. The CLT delivered key messages from the trial, whilst the SST completed ethnographic research in the field to uncover rumors and perceptions of the trial in the community. These ethnographic findings were shared with the CLT and addressed in targeted messaging to the community. Both the CLT and SST approached the communities in an egalitarian manner, by dressing modestly, speaking local dialects, and using **relatable** examples. Appreciation and understanding of the importance of interpersonal **relationships** and **respect** for the people, their customs, and traditions also played a large role in the CE program.

**Conclusion:**

These findings provide an in-depth understanding of how interdisciplinary community liaison and social science teams can work with a clinical team to strengthen trust. The four R’s suggest the ways in which trust relations are central to CE and confidence in vaccine trials, and could offer an approach to CE in vaccine trials.

## Background

Community engagement (CE) is an important dimension of disease control and preparedness interventions. Responses to the Ebola outbreak in West Africa between 2013 and 2016 understandably necessitated methods to engage the community in interventions to achieve effective disease control [1]. Amongst these response programs were the numerous Ebola vaccine trials implemented to prove an effective vaccine for Ebola virus disease (EVD). The EBOVAC-Salone and PREVAC vaccine trials’ CE programs were developed and delivered as a collaboration between the Innovative Medicines Initiative (IMI)-funded Ebola Vaccine Deployment, Acceptance and Compliance (EBODAC) and the Ebola vaccine (EBOVAC1) projects in Sierra Leone. This paper examines the relationships between trust, CE, and acceptance of a clinical trial.

### Community engagement in biomedical research

CE is used for a variety of capacities in biomedical research including participatory research and research evaluations [2]. Dicket et al. (2005) have proposed four main goals of CE: “enhanced protection, enhanced benefits, legitimacy, and shared responsibility” [ [3], p.1124]. How the public engages with biomedical research can influence the design, implementation strategy, level of uptake, and impact of disease control and prevention programs. As research in low and middle income countries (LMICs) increases, CE has become an increasingly important part of biomedical research methods and procedures, especially in programs involving vulnerable populations [4–6].

CE is important in biomedical research because it provides an opportunity for the researchers to understand the community’s needs and priorities [2]. CE may also help mitigate challenges faced by biomedical research. For example, CE activities can address public mistrust towards biomedical research by demonstrating respect for the communities involved in the research program [7, 8]. In addition to the potential benefits of CE, a review of evidence conducted by Attree et al. highlighted the potential for unintended negative impacts [9]; these include physical and mental exhaustion from the requirements of engagement that may not always outweigh the expected benefits [9] as well as further entrenching existing inequalities influenced by power and resource prioritization [10]. Furthermore, CE requires not only time and resources, but also researchers that are competent and capable of taking on the challenges of establishing this engagement with the community [11].

Some barriers to CE can arise from challenges in establishing equal relationships with the community. Biomedical research programs come with an imbalance in power between the researchers and the community, which could hinder CE if it is not acknowledged in a transparent and productive way [12]. This relationship can be particularly strained in marginalized communities where citizens may not have regular access to health care and the resulting benefits of biomedical research [12]. Additionally, existing relationship structures within a community may pose challenges in CE as participation can reflect existing “social hierarchies and economic or political divisions” [ [10], p.457].

To be successful many CE activities rely on mutually beneficial relationships and high levels of trust between the research program and the community [13–15]. In order to develop trust with the community, a study on the informed consent practices of a vaccine trial in Kenya used both external technical experts and local field assistants who were known to the community to convey information about a trial before it began [15]. Relationships are important to CE and biomedical research because they provide an avenue for researchers to understand the priorities, needs and context of a community [12]. The relationships between the community and the research program can also influence the community members’ perception of the research study and their willingness to participate [15].

### Community engagement in practice

MacQueen et al. (2015) have noted there were few guidelines available for the implementation and evaluation of CE programs in the context of global health [5]. Amongst existing guidelines, the Good Participatory Practice (GPP) Guidelines, developed by AIDS Vaccine Advocacy Coalition (AVAC) and the Joint United Nations Programme on HIV/AIDS (UNAIDS), provide guidance on engaging stakeholders in the design and implementation of clinical trials [16]. This document outlines six guiding principles (respect, mutual understanding, integrity, transparency, accountability, and community stakeholder autonomy) and various practices that could be used to develop beneficial relationships and engagement between trials and stakeholders [16]. Since being developed for HIV prevention trials in 2011, the GPP guidelines have been adapted and applied to engage stakeholders in various other research areas including tuberculosis and emerging pathogens [16–19]. While stakeholder engagement has become acknowledged as key to improving uptake and efficacy of trials, there has been limited documented evaluation on the actual implementation of the framework outlined by the GPP [17, 20–24].

Further to this limitation, Lavery et al. (2010) argue that there is little evidence on what makes CE effective in different contexts [14]. This highlights the gap in current discourse around CE and health interventions. Existing research on effective CE has revolved around participation in biomedical research such as vaccine trials. Much of this research centered on the social relationships influencing individual involvement [25] and the ethical implications of CE in biomedical research. For example, a study in Kenya found that CE enabled researchers to take opinions and concerns of participants into account at an early stage to adapt messages and identify effective methods to address any ethical issues that might emerge [26]. There is an opportunity to build on such work to use CE in the development of health interventions or clinical trials.

### Ebola in West Africa

Between 2013 and 2016, multiple countries in West Africa experienced outbreaks of EVD [27]. Beginning in Guinea, the virus spread to Sierra Leone in May 2014 through human-to-human transmission and continued for a sustained period [28]. Three months into the EVD outbreak, the World Health Organization (WHO) declared the EVD outbreak a Public Health Emergency of International Concern [27]. As of March 27th 2016, Sierra Leone experienced 14,124 confirmed, probable, and suspected cases and 3955 deaths due to EVD [29]. Interventions responding to the outbreak focused on containment strategies such as case isolation, contact-tracing and quarantine, and the training and implementation of sanitary funeral practices [30]. These non-pharmaceutical methods were emphasized because of the lack of experimental vaccines and treatment options at the beginning of the epidemic [31]. Two additional preventive interventions were also relevant in the 2014 Ebola response: infection control in health care settings and avoiding contact with bush meat and bats [32].

What was notable about the Ebola response in West Africa was the delayed response in effectively addressing the outbreak. Initial responses were lacking and may have been impeded by political or structural factors [33]. Other factors that served as barriers in controlling the outbreak and implementing interventions, included: mobile populations, lack of trust in governments, weak health systems and poor coordination [34]. These challenges were exacerbated by “inadequate communication strategy, misconceptions around the disease, ignorance of local culture and customs, and lack of involvement of local communities in the control strategies” which led to further distrust and reluctance from the communities [ [35], p.1]. For example, the association between some funeral rites and risk of EVD transmission [36] was poorly addressed until responders considered the cultural and social norms behind these behaviors and consulted local community members to implement safe and culturally respectful burial practices [1, 37].

In the early months of responding to the Ebola outbreak, the focus of many communication strategies was “sensitization, emphasizing that local populations lacked knowledge on Ebola and that ‘traditional practices’ spread disease” [ [38], p.2]. This focus on correcting misinformation did not engage the community in the process as an equal party. Other critics, explained that health messages failed to include practical information communities needed answered in real time such as “How do I manage a family of children, including infants and toddlers, in quarantine?” [39]. Hence, involving local communities and empowering community-based management were pivotal to containing EVD [39]. Laverack and Manoncourt emphasized this importance and found that intergovernmental agencies and non-governmental organizations (NGOs) learned from earlier mistakes and made an effort to increase CE later on in the response. These authors also argued for a reciprocal engagement approach that utilized bottom up communication strategies and incorporated local involvement [40].

One aspect of the West Africa Ebola response saw the fast-tracked implementation of trials to test candidate vaccines. Amongst these, the EBOVAC-Salone trial was set up with a CE approach, during the ongoing outbreak response, raising many challenges around conducting research in an emergency setting [41]. This paper will explore the CE approach implemented in two vaccine trials during and post the West Africa Ebola epidemic. The perspective taken will focus not on the ethical implications of CE, but rather on how it has been implemented in two case settings.

### Ebola vaccine trials in Sierra Leone

The EBOVAC-Salone vaccine trial was implemented to test a candidate Ebola vaccine in 2015, whilst the Ebola outbreak was still ongoing in West Africa. The trial was led by EBOVAC1 and supported by EBODAC. This trial studied a two-dose prime-boost vaccine developed by Janssen Pharmaceuticals and Bavarian Nordic in the Kambia District, one of the last districts in Sierra Leone reporting Ebola cases [42]. When setting up the trial, researchers considered the importance of understanding the socio-cultural context to build trust and engage with community members [38]. Because the Ebola outbreak was ongoing while these trials were developed, the climate, fear, and stigma around the disease and potential vaccine were a significant concern. Acceptance of the vaccine, which would be necessary for the clinical trials’ operation, could be influenced by multiple factors including: the social and cultural context of the population, perceptions of vaccine efficacy or safety, and trust or distrust in national and international authorities [43–45]. In the setting of the West Africa Ebola outbreak, resistance to outbreak response was not uncommon with reported cases of violence against health care workers [46, 47] and resistance to the ring vaccination trial in the area of Macenta in Guinea [48]. The two-dose nature of the prime-boost vaccine being studied added another layer of complexity to the challenges behind vaccine acceptance and compliance by necessitating every participant to receive the right vaccine at the right time. To address these challenges, the EBODAC project focused on communications, technology and community engagement to support the EBOVAC-Salone and later PREVAC clinical trials in Sierra Leone.

**Fig. 1:**
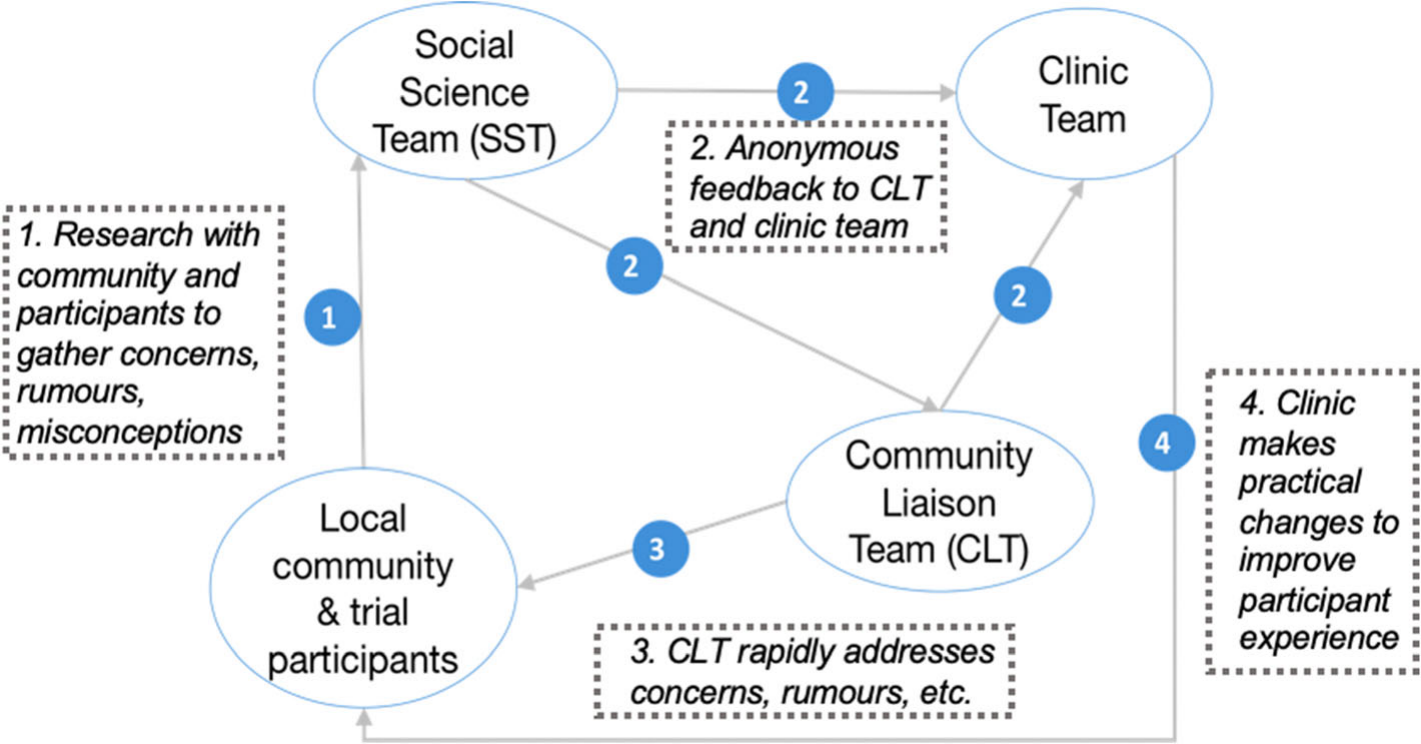
Community Engagement Structure . The trial’s community engagement structure, highlighting the interactions between the different parties. Source: EBOVAC trial community engagement diagram – Tom Mooney

The subsequent community engagement program implemented in the EBOVAC-Salone trials was shaped by the importance of understanding and tailoring communications to the local context [49] as well as previous participatory research techniques used in HIV prevention trials in Tanzania [50]. The use of a “community liaison system” [50] that connected the trial to the community was integral to the EBODAC/EBOVAC community engagement structure as demonstrated by Fig. 1.

In this structure, the trial recruited two local teams: a community liaison team (CLT) and a social science team (SST). The CLT, comprised of nine locally recruited staff employed by the University of Sierra Leone’s College of Medicine and Allied Health Sciences (CoHMAS) and two LSHTM supervisors. The team received background training on clinical trials and were responsible for implementing the CE strategy, monitoring rumors and concerns circulating in the community, and providing information about the trial at national and international levels. The SST was comprised of four locally recruited research assistants, a data analyst, a transcriptionist, and an LSHTM social scientist. This team explored community and participant perceptions and experiences of the study and the socio-cultural context. For example, in investigating trial participants’ reasons for joining the trial, the SST found community members were motivated by altruism, curiosity, access to health and the vaccine, and expectations of some benefit [51]. The SST conducted this ethnographic work independently from the recruitment efforts of the CLT, but these two teams informed each other as well as the clinical team to address rumours and misconceptions circulating in the community [38, 51]. The CLT was specifically trained as a connecting mechanism between the community and the trial. Notably, the SST was trained to be independent from the trial. The reality of how this was perceived will be discussed further.

The same CLT/SST approach was adopted in the PREVAC trial in Mambolo, Sierra Leone which started approximately two and a half years after the EBOVAC-Salone trial in May 2018. The PREVAC trial examines three different experimental vaccines which are compared with a placebo [52].

## Methods

We conducted a qualitative study to explore the experiences of conducting CE for the two vaccine trials that were set up during and after the 2014–2016 Ebola outbreak in Sierra Leone. This research used two case studies (i.e., the EBOVAC-Salone trial in Kambia and the PREVAC trial in Mambolo) to compare strategies and identify barriers and facilitators related to CE. Data were collected in Sierra Leone in May 2018. This was towards the end of the EBOVAC-Salone trial and in the first few weeks of the PREVAC trial.

One researcher (SD) conducted in depth, semi-structured interviews (IDIs) with EBOVAC-Salone and PREVAC CLT and SST members (15 participants). Participants were recruited using convenience sampling, based on team members’ availability. Interviews were conducted in English as all CLT and SST members were English-speakers. IDIs focused on the individual’s role in the trial and/or Ebola epidemic as well as their understanding of the CE model being used. Specific interview questions included: “What is your role on the trial? What are the key issues that arise during engagement? How do you think the community engagement you do addresses these concerns? Describe your experience working with the other trial teams.” Some of the interviewees were involved in both trials and were therefore able to compare their experiences of the two.

In parallel to the IDIs, one researcher (SD) conducted focus groups discussions (FGD) with community members living near to each trial site (2 FGDs) and with local leaders in Kambia (1 FGD). An FGD for local leaders in Mambolo was not conducted because of the unavailability of potential participants. FGDs were conducted with community members and local leaders due to time constraints and based on the advice from the SST that FGDs would be more conducive to free-flowing conversations in the community, especially when approached by a foreigner. FGD participants were recruited through a purposive sampling method; a local research assistant reached out to potential volunteers based on conversations and referrals from the SST. The local research assistant supported FGDs when language translation was necessary. These FGDs focused on participants’ experiences with the Ebola epidemic and with information activities around the trials. Specific questions included: “How did you receive information about the trial? Tell me about your experiences with the people delivering this information? What suggestions would you make to improve the community engagement around the trial?” Discussions and interviews were audio recorded with the permission of the participants and notes were taken by the interviewer. Interviews were transcribed (and translated where necessary). All interviews were anonymized to ensure confidentiality. After the first few interviews, themes emerging through initial thematic analysis were incorporated into the interview guide and explored in subsequent interviews. For example, when multiple interviews described the importance of ‘respect’ in the community engagement activities, the interviewer incorporated questions about how the trial teams demonstrated respect to the community in subsequent interviews and focus groups. IDI and FGD interview guides are provided in Additional file 1.

### Data analysis

Interview transcripts were imported into NVivo 12 Software. Another researcher conducted an overall thematic analysis of the data. As the data were analyzed, a working analytical framework emerged (the Four R’s) and was then used to identify similarities and differences as well as possible relationships in the entire data set [53]. Given staff time constraints, double coding of all interviews was not possible. However, one interview was double coded by another researcher with experience in qualitative research in Sierra Leone for validation of identified themes. This is an acknowledged limitation of this study. While a comparative analysis was not formally used, similar findings that align or do not align have been presented in the results. The results from this study are presented in two parts: how the community engagement program functioned (the roles of the teams) and why it functioned well (the Four R’s).

### Ethical considerations

Ethics approvals were obtained from the Sierra Leone Ethics and Scientific Review Committee and the London School of Hygiene and Tropical Medicine (LSHTM). All study participants were volunteers, received participant project information sheets, provided written consent and agreed to have their anonymized quotations included in publication. FGD participants received a small travel allowance of 20,000 Sierra Leonean Leones (approximately £2) and light refreshments. This was in line with ethical approaches within the trials and the Sierra Leone research community.

Findings are presented as quotes or paraphrased descriptions using the following notation: CLTxxx and SSTxxx refer to IDI participants with an anonymized identification number while FGDxxx is attributed to a particular focus group but not a specific participant.

## Results

The findings of this study provide insight on the delivery of a community engagement programme primarily from the perspective of the individuals delivering it. The CLT felt their role was to explain the trial and its importance to the community, recruit participants and to address any rumours or misconceptions the community had about the trial (CLT004). They did this through a variety of activities including one-to-one stakeholder meetings, group area meetings, public performances and radio jingles (CLT002, CLT009, CLT010). The SST served two functions. The first was to support the trial through power mapping to understand the community’s leadership structure, community household mapping to collate geographic locations, and ethnographic research to explore rumours and concerns circulating in the community of interest (SST001, SST002, SST004). The second was to produce independent research on the social aspects of the trial to understand what factors led people to participate in the trial and their experiences as participants (SST001). Finally, the Participant Advisory Group (PAG) was a group of trial participants that served in an auxiliary capacity to the trial. They held their own meetings to discuss grievances and concerns and reported their priorities for action back to the trial (CLT004).

SST and CLT members that were interviewed brought up a number of challenges faced in recruiting participants for the trial. It was often difficult to locate people because they did not know their addresses or because they had businesses or farms that kept them out of the house all day. Due to the community members’ busy schedules, the CLT would conduct their house-to-house visits early in the morning or late in the evenings and would ask organization leaders to book times that worked best for their members for area meetings (CLT002, CLT003, CLT004, CLT007). From the perspective of the CLT, many in the community were unwilling to listen to anything relating to Ebola because of residual fear of the disease based on their community’s recent experiences with it (CLT001) or could be unwilling to attend meetings or discuss the trial because *“people’s minds are very fixed from what they told them [during the outbreak]”* (CLT008). This perception of an unwillingness to learn about the experimental vaccine was verified during FGDs in Mambolo and Kambia (FGD001, FGD002). Local community leaders in Kambia explained that they did not trust this experimental vaccine when they first heard of it because of the experiences they had during the outbreak where they were also told there was no medicine to cure Ebola (FGD002). These were some of the sources behind the hesitancies that the SST and CLT would need to address.

**Fig. 2:**
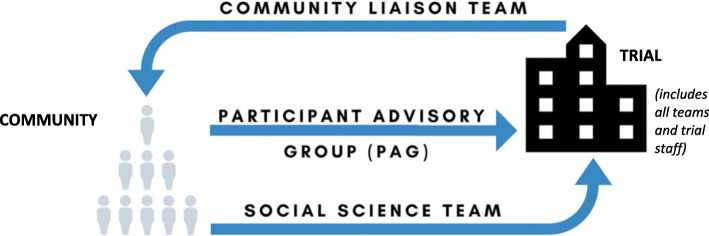
“Bridges of Information**”.** The flow of information in the community engagement program of the vaccine trial based on descriptions from interview participants

Interestingly, both teams described themselves as a bridge between the trial and the community. This could be because both teams felt they were a mechanism that ensured that the voices of the community were raised and heard by the clinical trial team. While both teams viewed themselves in this similar capacity, they carried out this role in different ways. While the CE program structure had a few mechanisms for feedback (Fig. 1), CLT and SST members typically described a flow of information that is represented in Fig. 2.

This section describes four characteristics of the CE program and the dynamic presented above that were uncovered throughout the interviews.

### The Four R’s

Trial team members expressed positive impressions of their work in the community, citing the high number of trial participants as evidence of their success. When asked about how the CE work of the trial had been successful, both individual team members and FGD participants pointed to the importance of trust. As one CLT member said:

*If people do not have confidence in you, […*] *you will not succeed. Yes, the first thing is confidence building – trust. People need to have trust in you that presented the message. If they do not have trust in you, even when you present the message, they will sit down and listen to you, [but] these people do not accept the messaging.*. *.* (CLT008).

Throughout the interviews, four concepts emerged relating to the activities of both the CLT and SST: reciprocity, relatability, relationships, and respect. These were not formal principles that staff were trained on, but they helped establish the trust between the community and the trial staff through their delivery and influenced the success of the CE.

### Reciprocity

There were several ways through which this CE model established a reciprocity that created effective engagement. The first was the CLT-SST feedback loop that allowed for any issues in the community to be both heard and addressed. Another avenue that created this reciprocal relationship was the exit interviews conducted by SST members at the clinic. These conversations, that occurred at the end of a participants’ visit to the clinic, allowed the SST to bring information from the trial participants back to the trial – both the CLT and clinic staff. This forum was also appreciated by the community members:

*One thing I love about them whenever you go there for the marklate [vaccine] immediately after taking the marklate [vaccine] they have an interview room where they will take you and whatever you say there is secret. They give you confidence to ask questions …* (FGD002).

This opportunity to provide confidential feedback, including potentially negative feedback or criticism, on their experience built trust among the community members towards the trial. This sentiment also echoed an SST member’s comments on how their team provided an aspect of care – an opportunity for feedback and follow up – typically missing from the hospitals and health care system in Sierra Leone (SST001). Trial participants were also able to discuss and provide feedback on the trial through the established PAG. This group contributed to the trial by bringing participants together to discuss with each other their grievances. This group was completely independent of the trial, with the ability to bring certain issues to the trial team’s attention.

Reciprocity could also be seen in the format of area meetings held by the CLT. At these meetings, community members had an opportunity to ask their own questions after the trial staff presented their information. In addition to answering questions, CLT members would interact with meeting attendees to keep them engaged and interested. A CLT member explained:

*We ask them questions – how are they getting the message. We tell them ‘ask us questions.’ If they don’t ask us we say okay we will ask you. We interact.*. *. We will ask them the questions.*. *. How many marklates [vaccines] are they giving you? They tell us. If they make a mistake we correct them.* (CLT003).

By consulting local authorities, the trial teams were able to improve the trial procedures to make them more acceptable to participants. One challenge they faced in May 2018 was how the holy fasting month of Ramadan would affect trial participation. A CLT member explained that they approached this challenge by reaching out to the religious leaders for advice on what they thought the appropriate response to questions about what the religion says about observing the month of Ramadan and participating in a trial that involves injections or drawing blood (CLT010). This inclusion demonstrated both a respect (another one of the four principles) for local leaders and beliefs as well as reciprocity as the community contributed directly to the functioning of the trial. The overlap between reciprocity and respect was also clear in the practice of providing refreshments during meetings. An SST member that observed area meetings in both trials described the importance of these:

*This model of providing the refreshment for people plays a very big role because we have this type of reciprocity. If I give you, you should be able to give me in return. Whichever way - I am sacrificing my time, I am sacrificing my business, I am sacrificing my other economic activities to come and listen to you to help to promote your agenda. What do I stand to gain in return?* (SST001).

This concept of exchange is not unusual or unexpected [51], but it could be a delicate matter to apply in a way that does not coerce or bribe research participants. This CE approach could be adopted in future trials to support finding the balance between these issues.

### Relatability

Another theme that became apparent in interviews was the relatability of the trial team members with the community members. Most of the EBOVAC-Salone and PREVAC CLT and SST members were recruited locally. The CLT and SST staff could relate to their target audiences as they shared the same cultural and social norms, appearance, language, and terminology. The first impression from appearance was agreed to be an important first step in being able to engage with the community. A CLT member explained the importance in dressing modestly because:

*If you dress as if you are the president of this country, some of these stakeholders will not even talk to you. They will say that these are the people that are eating the country’s money so we see no reason for us to listen to them*. *.*. *That’s why you need to put yourself in that moderate manner so that maybe when you go there they will think that all of us, we are equal.* (CLT004).

Another form of relatability comes from using local institutions and infrastructures for the project. For example, the use of a local town crier demonstrated respect for that institution while reaching community members in a way to which they were accustomed. In using these existing systems of communication, it was vital to consider language. A common language (Krio, Temne, Susu or Fula depending on the setting) not only allowed for conversations to take place, but also facilitated a better understanding, comfort, and trust between the two parties (CLT004).

In addition to speaking the same language, CLT and SST members needed to account for some of the scientific terminology used to explain the trial. An SST member highlighted the need to break down complicated or scientific terminology into relatable terms (SST001). One example of this that was observed throughout the interviews was the use of the word *“marklate”* instead of “vaccine” or “injection.” Between 1988 and 1990, there was a national campaign to eradicate polio from the country [54]. This large-scale campaign and the “polio marklate” were relatable experiences for the community members. Because the people were more familiar with that word, it became a term frequently used by both trial staff and community members to refer to the experimental Ebola vaccines. Another example was that there was no local term for meningitis. Instead, a CLT member said they would explain the condition and its symptoms without calling it ‘meningitis’ as this was not a familiar word (CLT003). Employing appropriate language and terminology could be a considerable challenge when discussing nuances in a ‘naïve community’ with no experience in biomedical research.

Relevant examples and simple metaphors were another way the CLT members related to community members in order to convey their message. When community members questioned the extensive screenings required before administering the vaccine, CLT members explained how a doctor in a hospital would run tests to confirm diagnosis if they were ill because this was an experience with which they were familiar (CLT005). A vaccine trial was a new experience in the community and so the CLT needed to explain that they were testing the vaccine being administered. A CLT member said they would mention existing vaccines that the community knew and would explain that these vaccines went through similar processes around the world before they could be dispensed in their own communities (CLT010).

Focus group participants confirmed that the CLT members gave clear information with a relatable football metaphor that they could understand (FGD002). One SST member described the value of this strategy to justify why staff members were not taking the experimental vaccine:

*People want to hear not just explanations, but practical examples. That will force them to believe. For example, you set up the scenario of a footballer and a referee. In a football match, you cannot be referee and at the same time, play the match. What will happen if you play a foul that leads to a penalty kick – would you blow that whistle? Any time this kind of example is made, you will see people laugh. You will see them nodding their heads, nodding their heads in acknowledgement.* (SST001).

Finally, this relatable demeanor and language was complimented with visual aids. To respond to the concerns over blood sampling, the CLT brought the test tubes used at the clinic to the area meetings. They filled a test tube with water and poured the tube’s contents into their hands to demonstrate the small volume that would be collected. In each FGD, participants recalled this display when asked about the type of information they received at area meetings.

### Relationships

The next principle highlighted throughout the interviews was the emphasis on relationships in these communities. Both staff and community members expressed the value of family and friend relationships in Sierra Leone. Two CLT members explained their roles would not have been as effective had they been strangers to the local community because the fact that the people knew and trusted them was important in delivering their messages about the trial (CLT005, CLT003). This high value on relationships was why it was important for trial staff to be recruited locally. Local residents hired for CE work had already established both relationships and reputations within the community that primed them for effective trial message delivery. Multiple SST members agreed that local staff was important for the trial to succeed (SST001, SST004).

An additional benefit of local staff was the connections they brought. A CLT member explained that their *“relationship between we the community liaison and the paramount chief, the heads of different areas, is very cordial because most of the stakeholders, they are our parents”* (CLT008). This was acknowledged by community members as well and given as one of the reasons they trusted them. In Mambolo, an FGD participant explained:

*Some of them are related to stakeholders here, some came from ruling houses, so as a matter of fact, there is that trust […*] *that confidence in them and like the previous speakers said, some of them are our former school pupils and some of them have attained higher education. So, with all of that, there is that confidence and trust to accept whatever information they brought us.* (FGD001).

This speaker mentioned the value of not only the CLT members’ relatives’ reputations but also their own. Growing up and interacting with the community throughout their lives gave them the credibility to approach their neighbors for the purpose of the trial.

This idea was echoed in a Kambia FGD as well:

*We trust them because they are native born of Kambia and we know them. We know that they cannot take us somewhere where we will lose our lives... Everyone is related to each other and they cannot contribute to trouble for each other. Brothers and sisters always bring good thing for each other. So, that is why once I see them, I am always ready to listen to them.* (FGD002).

This agreement featured another example of the strong sense of family and relationships in these communities that was apparent in observing community area meetings and FGD dynamics. This attitude and regard for others as “uncle, auntie, son, daughter” demonstrated both respect and a high regard for interpersonal relationships. For example, community members referred to elders or local authorities as “ma” or “pa” and to younger people as their own children. As one CLT member said, *“When the people see me, they build up confidence because I am their son”* (CLT008).

### Respect

Respect is interwoven throughout the activities and examples of the other three principles and is an underlying factor in any engagement. The CLT and SST members needed to trust and respect the work of their colleagues. This was a common challenge in the beginning of the trial, with some SST members describing an initial tension between the CLT, SST, and clinical teams. One SST member explained this tension as a result of providing feedback on aspects of the trial that could include the behaviors of trial staff such as an error made in the clinic or a confusing statement described at a community meeting (SST005). These comments on actions trial staff did or did not take could then be perceived as a personal attack (SST001, SST002). Addressing this issue took time and patience; another SST member explained that more frequent training sessions and checkpoints between the different teams mitigated the tension (SST003). The mutual respect that eventually developed at these meetings allowed the teams to move forward together.

Trial team members stressed the importance of demonstrating respect to the community members in the areas they worked. A CLT member provided this scenario to illustrate the overlap between relationships, reputations, and respect:

*First of all, one thing about community people, if you are indigene of this place, make sure you have respect for people. You need to have respect… Now when you have respect, here is a village area, so when you greet somebody you do not call them by names, you call them my uncles, my auntie, my grandmother. With that respect, when they see you my son, my daughter, have this bucket go and find water for me. My son, please come and help me pound this rice. My son, today I have work in my farm, please go and help me. From that, people build up confidence in you. That you are a son or a daughter that have respect for them. (CLT008).*

The CLT members believed they were respectful by answering the questions of community members and through greeting people in the typical custom with handshakes instead of handwaving from a distance (CLT009, CLT010). Community members and local leaders agreed that these behaviors and how the staff carried themselves showed that the trial teams respected them. In one FGD, community members explained that a disrespectful person would not be accommodating and approachable, but the CLT and SST staff were always polite and encouraging (FGD002). Another participant expressed an appreciation for the way trial team members were receptive to any comments they wanted to share and would respond in a respectful tone and manner (FGD002).

In addition to this behavior at the area group meetings, community members felt the time trial team members would take to visit them at their own homes to answer questions demonstrated their respect (FGD003). Ensuring confidentiality in these one-on-one conversations became another way to show respect for the community members. When an SST member heard a rumor or concern that needed to be corrected, they only reported the area where the rumor was circulating rather than the individual who conveyed it (SST003). This respect for privacy encouraged community members and trial participants to continue to be open with their feedback or criticism of the trial when speaking with the SST.

When asked if the community members respected the trial team members in return, there was a resounding unanimous agreement. When questioned why they respected the trial team members, one participant aptly reported:

*Because this is just like if you don’t want yourself to be slapped by someone, don’t slap anyone [laughing …*]. *So if you respect somebody, somebody will respect you in return. They have already given us our own respect, why don’t we give them the same respect?* (FGD002).

Respecting the existing power hierarchies and structures of the community was imperative for the program because of the high respect community members had for their local authorities and leaders. The CLT understood that it was crucial for important stakeholders to be actively involved in order for the rest of the community to be willing to listen (CLT008). An SST member further explained that *“the local authorities must not be seen to be sidelined. They must be seen to be taking the center stage, because our people impose high trust on these local authorities”* (SST001). This respect for local leaders also aided the trial when a key leader of Kambia became a trial participant. Community members in both Kambia and Mambolo cited this example as one of the reasons they believed that the trial was not malicious. Seeing an individual of such high status and regard taking the experimental vaccine had a noticeable influence on the perceptions of the trial.

The trial team members exhibited their respect for the local leaders not only by reaching out to them first, but also by having them set the times and terms of their meetings. They also made sure to acknowledge the relevant customs and traditions in the community. One CLT member gave the example of bringing a small token to the chief as a sign of respect to him and to the local customs (CLT010). While this custom would be a sign of respect in this community, in some settings, it could also be misconstrued as a bribe. This is one example of a challenge in maintaining a balance between respecting customs and practicing appropriate research ethics.

## Discussion

This research explored the application of a CE program in two Ebola vaccine trials in Sierra Leone, primarily through the perspectives of the people implementing it. While this study set out to find barriers and facilitators to such CE, it mainly found patterns in qualities that served as facilitators in this specific CE program. Similar to other biomedical research programs with CE components, this collaborative model delivered by the EBODAC/EBOVAC partnership allowed the trial to consider community perceptions and adapt its practices accordingly [4, 8, 15, 26, 50, 55]. While the EBOVAC-Salone and PREVAC trials were implemented in different time periods and geographic settings, participants felt the distinguishing factor was that EBOVAC-Salone was one of the first clinical trials of its kind in the country, not that it was initiated during an ongoing outbreak.

If CE is as pivotal to epidemic recovery, preparedness, and response as Wilkinson et al. [1] argue, it is useful that this study reveals four characteristics (reciprocity, relatability, relationships, and respect) that address complex social relations between trialists and community members. These findings are in line with the literature that point to relationships and respect as key aspects of a successful CE program. Analyzing the EBODAC/EBOVAC CE approach used in the EBOVAC-Salone and PREVAC trials in Sierra Leone has shown that these two principles, along with relatability and reciprocity, could influence the success of a CE program. Whilst these characteristics have been identified as important in other literature [7, 10, 12, 15, 16, 26, 50], this study has revealed the importance of using all four in conjunction. Additionally, this study considered the implementation of the CE model in an outbreak and post-outbreak setting and found that both drew on the same four principles.

From the narrative of the participants, the inclusion of the independent SST and CLT served in different capacities as a bridge between the trial and the community. While this overlap in the perceived role of the two teams was unintentional, this bidirectional flow of information and conversation was integral to reciprocity. The idea of a reciprocal link between a trial and a community is not a new one. Community advisory boards as well as other participatory research models are common in biomedical research [26, 50] and are predicated on a principle of reciprocity by requiring a form of direct involvement from the community. For the trials examined in this study, the separation of the SST and CLT is what fostered an equal and reciprocal relationship; for example, by allowing for independent and confidential exit interviews conducted by the SST.

To communicate effectively with the community, these teams used local languages, terminology, knowledge of social norms, and examples to be relatable and easily understood by the community. This quality of relatability builds off of the principle of mutual understanding outlined by the GPP [16]. Additionally, the recruitment of staff from the local communities where the trial would be implemented influenced their work during the trial but was also influenced by existing power dynamics and structure. Some studies have described the challenges of avoiding an asymmetrical relationship of power between the community and the trial [7] or of understanding the distribution of power and the social hierarchical structure of a community itself [10, 12]. Previous research has shown the importance of positive relationships on a community’s perception of biomedical research programs and how CE can be used to demonstrate respect and address public mistrust of biomedical research [7, 12, 15]. The relevance of power was also clear in the emphasis on respect. The SST and CLT had to respect such power structures, for example by communicating via local leaders, in order to be heard by or have access to the community. Previous literature has found that demonstrating respect through CE has not only an ethical importance but also aids in the development of trust and encourages participation [7]. This is in line with the GPP highlighting respect as one of the guiding principles in stakeholder engagement [16].

While these four concepts could aid in applying CE in biomedical research naïve communities, they also raise important considerations to be addressed. Though it was not explicit in the findings of this study, the high value of relationships in this CE program could be problematic. For example, understanding community ‘leadership’ and power dynamics can pose challenges in the identification of and respect given to key ‘stakeholders’ in the community [38]. In the community of Kambia in Sierra Leone, it is notable that these power structures were not necessarily straightforward and it was imperative to understand these nuances in order to effectively engage with the community [38]. There is some indication from this study that connections to family or previous relationships with local leaders were influential to the teams’ work and rapport with the community. It may be useful in the future to consider how this could then influence the dynamics of power, representation, and privileged access to staff or resources***.***

These considerations can be further expanded to the use of CE in general. There is a great deal of momentum behind supporting CE in biomedical research, and in outbreak response settings as well. However, the unusual circumstances and challenges of emergency settings must not be overlooked or generalized in attempting to fit a CE program to the response. Many in the field have already pondered questions such as: at what point do trialists engage the community and to what degree? How do we balance what may seem to be conflicting priorities of clinical care, biomedical research, and open communication? How do we manage power and fairness in an emergency health setting that is inherently unfair? How does CE interact with existing power imbalances or resource inequities? [16, 23, 56, 57] These questions and challenges cannot be addressed with a ‘one size fits all’ model; further research is needed to look at the impact of CE that incorporates the Four R’s on the process, priorities and power of engagement.

### Limitations

The scope, both in time and resources, of this study served as a limitation affecting both sample size and data collection. While data saturation was achieved among IDI participants (demonstrated by the repetition among responses), it could have been useful to speak to a wider audience of community members and trial participants to gain a better understanding of their perceptions of the trial and its CE activities. Additionally, it is possible that participant bias may have been present in some of this study’s participants’ responses given their roles as staff on the EBOVAC-Salone and PREVAC trials. Because of their positions, participants may have been inclined to answer questions in a more positive light.

Another limitation not foreseen was the delay of the PREVAC trial. The trial launch was originally planned for early 2018 but did not actually roll out until just a few weeks before data collection began. This delay meant that CLT members and Mambolo community members did not have as much experience with the trial or messaging as was expected. Additionally, because individual interviews were conducted in English, some nuance in responses may have been lost that would have been differently expressed in a native tongue. Another limitation came from the phrasing of certain questions. Interview topic guides were reviewed with the assistance of the SST in Sierra Leone to check for technical words or phrases that would not be clear for interview participants. Despite this, some interviews still had the occasional misunderstanding. In some instances, rephrasing a question or providing examples of potential answers may have biased the response through unintentional leading of the participant. Future studies would benefit from testing the questions a priori and using local languages during interviews.

## Conclusion

In conclusion, the CE approach delivered through the EBODAC/EBOVAC partnership depended on trust established between the teams and community members that was reciprocal, relatable, relational, and respectful. These are qualities that have been mentioned in the existing literature on CE, but not as one comprehensive framework that could foster trust and therefore effective engagement. Furthermore, the ‘Four Rs’ could also be further developed as a tool to benchmark effective community engagement; to do so would require additional work to ensure the creation or adaptation of appropriate indicators for monitoring. While some of these findings may be significant to the setting in Sierra Leone and the Ebola vaccine trials or may be influenced by some of the limitations in this study, it is hoped that these Four R’s could facilitate CE in other settings and strengthen disease preparedness activities. Furthermore, gaps in the literature also call for more in-depth research to be conducted on effective models of CE for both global health emergency and disease preparedness settings.

## Supplementary information

**Additional file 1.** IDI and FGD interview guides.

## Data Availability

In order to protect the privacy of research participants, the raw qualitative data for this project will not be publicly available.

## Abbreviations

CE: Community engagement
CLT: Community liaison team
EBODAC: Ebola Vaccine Deployment, Acceptance and Compliance
EVD: Ebola virus disease
FGD: Focus group discussion
IDI: In-depth interview
IMI: Innovative Medicines Initiative
PAG: Participant advisory group
SST: Social science team
