# Supplementary material for: Lessons learned from engaging communities for Ebola vaccine trials in Sierra Leone: reciprocity, relatability, relationships and respect (the four R’s)
Source: BMC Public Health. 2019 Dec 11;19:1665. doi: 10.1186/s12889-019-7978-4 (PMC6907283; doi:10.1186/s12889-019-7978-4)
Supplement: Supplementary file 1 — Additional file 1. IDI and FGD interview guides. [file 12889_2019_7978_MOESM1_ESM.docx]

# **Appendix 1**

## In-Depth Interview Topic Guide – Trial Team Members

Part 1: Background

*For this first part, I am going to ask you some questions about your background and experiences and your current role on the EBOVAC trial. I also have a few questions about your experiences with the Ebola epidemic.*

1. What is your role?
2. What has been your previous experience in a similar role?
3. Tell me about the community that you live in?
4. How long have you lived in this community (Kambia/Mambolo)?
5. What was your experience of the Ebola epidemic?
   1. Did you have a role in the response?
   2. If so, what was it? What did you do?
6. How does the community talk about the epidemic now?
   1. Where do people think it emerged from?

Part 2: Understanding Community Engagement

*This next section is more about being a part of the community liaison/social science team and the work that you do.*

1. What do you understand by “community liaison”?
   1. What does “liaison” mean to you?
2. What do you understand by social mobilization?
   1. What does mobilization mean to you?
3. What experiences have you had with community engagement before the trial?
   1. (probe: NGOs, epidemic response)
4. Who are the key people that you engage with?
   1. What does this involve?
   2. What is successful about this engagement?
   3. What are some of the barriers or challenges to this engagement?
5. Who are the people you are unable to engage with?
   1. Why are you unable to engage with them?
   2. (probe: time, access, priorities)
6. What are the key issues that arise during engagement?
   1. (probe: rumors, concerns, questions about the trial)
   2. (probe: Ebola, vaccine science)
      1. What do people understand about how Ebola is transmitted? What do they understand about the role of animals/bushmeat in Ebola?
      2. Are you still correcting misinformation about the epidemic?
7. How do you think the community engagement you do addresses these questions or concerns?
   1. What works?
   2. What would you change?

Part 3: Model of Community Engagement

*This final section about how this model of community engagement works in the EBOVAC trial and what you think about the program overall.*

1. Tell me about your experience of working with the social science team?
   1. How has this contributed to your engagement activities?
2. What suggestions would you make to improve community engagement?

##

## Interview Guide – Community Focus Group Discussions

Part 1: Background & Experiences Around Ebola Epidemic

*For this first part, I just want to know a bit about the community you all live in and what your experiences with the Ebola epidemic were like.*

1. Tell me about the community that you live in (Mambolo/Kambia)?
2. What was your experience of the Ebola epidemic in this community?
   1. When did you first hear about Ebola?
   2. Can you explain to me how and why people became sick with Ebola?
      1. (probes: bushmeat, contact with animals, person-to-person)
   3. How did your community experience Ebola?
3. How did you get information about the Ebola epidemic?
   1. (probes: social mobilization during epidemic, health information on radio/tv/posters, community engagement for the trial)
4. What has been your experience with the EBOVAC/PREVAC trial?
   1. How or why did you become involved with the EBOVAC/PREVAC trial?

Part 2: Community Engagement in the EBOVAC/PREVAC Trial

*This section is about how you learned about Ebola, vaccines, and the trial.*

1. Tell me about your experience with **information activities** around the trial?
   1. What information have you received?
      1. (probe: transmission, zoonotic aspect, environmental control)
      2. How did you receive this information?
   2. What has been successful about these activities?
   3. What have been the challenges?
   4. Tell me about your experiences with the people delivering this information?
      1. Do you trust them? Why do you trust them?
2. What impact do you think the trial has had on you and/or the community?
3. What suggestions would you make to improve community engagement around trial?

Part 3: Understanding Community Engagement

*In this part, we will be talking about community engagement.*

1. What do you understand by “community engagement”?
   1. What does “engagement” mean to you?
2. What do you understand by “community liaison”?
   1. What does “liaison” mean to you?
3. What do you understand by social mobilization?
   1. What does mobilization mean to you?
4. What experiences have you had with community engagement before the trial?
   1. (probe: NGOs, epidemic response)
